# Genetic risk factors of Alzheimer’s Disease disrupt resting-state functional connectivity in cognitively intact young individuals

**DOI:** 10.1007/s00415-023-11809-9

**Published:** 2023-06-26

**Authors:** Ludmila Kucikova, Jianmin Zeng, Carlos Muñoz-Neira, Graciela Muniz-Terrera, Weijie Huang, Sarah Gregory, Craig Ritchie, John O’Brien, Li Su

**Affiliations:** 1 Department of Neuroscience, Faculty of Medicine, Dentistry and Heath, Sheffield Institute for Translational Neuroscience, University of Sheffield, 385a Glossop Road, Sheffield, S10 2HQ SY UK; 2 Insigneo Institute for in Silico Medicine, University of Sheffield, Sheffield, UK; 3 Sino-Britain Centre for Cognition and Ageing Research, Faculty of Psychology, Southwest University, Chongqing, China; 4 Department of Psychiatry, School of Clinical Medicine, University of Cambridge, Cambridge, UK; 5 Edinburgh Dementia Prevention, Centre for Clinical Brain Sciences, University of Edinburgh, Edinburgh, UK; 6 Ohio University Heritage College of Osteopathic Medicine, Ohio University, Athens, OH USA; 7 School of Systems Science, Beijing Normal University, Beijing, China; 8 Scottish Brain Sciences, Edinburgh, UK

**Keywords:** Functional connectivity, Large-scale networks, Functional neuroimaging, Alzheimer’s disease, Risk factors, Cognition

## Abstract

**Background:**

Past evidence shows that changes in functional brain connectivity in multiple resting-state networks occur in cognitively healthy individuals who have non-modifiable risk factors for Alzheimer’s Disease. Here, we aimed to investigate how those changes differ in early adulthood and how they might relate to cognition.

**Methods:**

We investigated the effects of genetic risk factors of AD, namely *APOEe4* and *MAPTA* alleles, on resting-state functional connectivity in a cohort of 129 cognitively intact young adults (aged 17–22 years). We used Independent Component Analysis to identify networks of interest, and Gaussian Random Field Theory to compare connectivity between groups. Seed-based analysis was used to quantify inter-regional connectivity strength from the clusters that exhibited significant between-group differences. To investigate the relationship with cognition, we correlated the connectivity and the performance on the Stroop task.

**Results:**

The analysis revealed a decrease in functional connectivity in the Default Mode Network (DMN) in both *APOEe4* carriers and *MAPTA* carriers in comparison with non-carriers. *APOEe4* carriers showed decreased connectivity in the right angular gyrus (size = 246, p-FDR = 0.0079), which was correlated with poorer performance on the Stroop task. *MAPTA* carriers showed decreased connectivity in the left middle temporal gyrus (size = 546, p-FDR = 0.0001). In addition, we found that only *MAPTA* carriers had a decreased connectivity between the DMN and multiple other brain regions.

**Conclusions:**

Our findings indicate that *APOEe4* and *MAPTA* alleles modulate brain functional connectivity in the brain regions within the DMN in cognitively intact young adults. *APOEe4* carriers also showed a link between connectivity and cognition.

**Supplementary Information:**

The online version contains supplementary material available at 10.1007/s00415-023-11809-9.

## Introduction

Alzheimer’s Disease (AD) affects an increasing number of people globally with no effective disease-modifying therapy currently available. Literature indicates the presence of brain changes related to AD decades before the onset of observable clinical symptoms [1]. Investigating early disease biomarkers is, therefore, crucial in prevention efforts, in understanding the nature of the disorder and for developing potential therapeutic interventions which might include risk factor modification.

Neuroimaging data has widened our understanding of the pathophysiology of AD and demonstrated the potential of developing neuroimaging-related biomarkers [2] including those based on network connectivity [3]. Resting-state networks have been observed to be already disrupted in the early stages of AD and deteriorate as the disease progresses [4]. Such changes are also present in healthy individuals with non-modifiable risk factors associated with AD in similar networks and brain areas across adulthood [5]. Notably, most studies report that connectivity changes already occur in middle-aged *APOEe4* carriers. However, how early those AD-related changes occur prior to middle age is still not clear. Further robust evidence from other genetic risk factors in early life is also lacking.

The evidence from published literature focuses on the Default Mode Network (DMN) analyses due to its spatial overlap with amyloid distribution and tau pathology [6]. Brain areas of the DMN exhibit coherent activity when the individual’s focus of attention is not attributable to specific stimuli. The DMN activity has been linked with internal processes such as mental stimulations, introspection, or autobiographical retrieval and might be heterogenous across the network [6].

The DMN seems to be consistently disrupted in individuals who have genetic risk factors for AD; yet the directionality of those changes is inconclusive [5]. Similarly, the areas of the DMN display different functional connectivity in healthy individuals with AD-related amyloid [7] and tau pathology [8]. Other resting-state networks that show involvement in at-risk populations include the executive networks [9, 10] and the salience network [9–11]. Connectivity in resting-state networks also correlated with the performance on a set of cognitive tasks in individuals with AD-related pathology. For example, alterations in executive functions and inhibition control assessed as performance on the Stroop task in cognitively healthy individuals with amyloid pathology were linked with a decrease in DMN connectivity [12].

In this study, we have investigated the differences in resting-state functional connectivity between young carriers of genetic risk factors for AD and non-carriers using a candidate-gene approach (i.e., *APOE* and *MAPT* genes). These genes were selected as *APOEe4* is consistently reported as a high-risk factor for AD [13], and *MAPT* is involved in tau production, which is aberrantly aggregated in AD and related tauopathies [14].

Additionally, the effect of connectivity and genotype on the Stroop task performance is investigated. Here, the Stroop task was chosen because it is widely used in both clinical and research settings as a reliable benchmark cognitive test in healthy young adults, for whom most clinical neuropsychological tools are unable to detect a change. It assesses selective attention, inhibition control, processing speed, cognitive load, and brain automaticity. Furthermore, previous evidence suggests that the Stroop effect is a robust predictor of neurodegeneration [15].

## Methods

### Study sample

A total of 392 young and cognitively healthy self-declared Chinese Han college students were recruited as an extension of the PREVENT-Dementia study [16] from Southwest University in Chongqing, China. Research ethics was provided by Southwest University’s local ethics committee. All participants provided informed written consent. Self-declaring as Han Chinese was part of the inclusion/exclusion criteria of the recruitment.

All participants provided a saliva sample for DNA genotyping. The genotypes for *APOE* and *MAPT* (rs242557) were determined by the Mass Array system (Agena iPLEX assay, San Diego, United States). For the purpose of this study, the study population was dichotomised based on (1) the presence of at least one copy of the *APOEe4* allele (“*APOEe4* carriers”); (2) the presence of the *MAPT A* allele, so that *MAPT A* and *MAPT AG* were grouped into one group (“*MAPTA* carriers”); (3) the presence of the *MAPT G* allele, so that *MAPT G* and *MAPT AG* were grouped into one group (“*MAPTG* carriers”). Based on the literature [17], we predicted that *APOEe4* carriers and *MAPTA* carriers would have an increased risk for dementia in late life.

A subset of 155 participants underwent neuroimaging. Anatomical images from participants with poor segmentation quality and functional MRI images with poor acquisition quality were excluded resulting in a final dataset of 129 participants for the subsequent analysis. Demographic information is summarised in Table 1. There were no significant differences in gender, age, and education between the groups.

**Table 1:** Summary of the demographics for the *APOEe4, MAPTA*, and *MAPTG* carriers and non-carriers. Family history was determined by grandparents’ diagnosis of any form of dementia because none of the participants’ parents have been diagnosed

	*APOEe4*		*MAPTA*		*MAPTG*	
	Carriers (n = 27)	Non-carriers (n = 102)	Carriers (n = 97)	Non-carriers (n = 32)	Carriers (n = 83)	Non-carriers (n = 46)
Gender (f/m)	16/11	54/48	53/44	17/15	48/35	22/24
Age (y ± SD)	19.6 ± 0.98	19.6 ± 0.86	19.6 ± 0.9	19.5 ± 0.83	19.5 ± 0.87	19.6 ± 0.93
Education (y ± SD)	12.9 ± 0.55	13.1 ± 0.59	13.1 ± 0.59	13.0 ± 0.54	13.1 ± 0.58	13.1 ± 0.59
Family history (n)	2	7	7	2	7	2

### Neuroimaging acquisition

MRI imaging data were acquired on a 3 T Siemens whole-body scanner at Southwest University. Participants underwent brain scans at rest while instructed to keep their eyes closed and not to think about anything specific. Resting-state echo planar images were obtained (35 slices; slice thickness: 3 mm, TR/TE: 2000 ms/30 ms, flip angle: 80°, FOV: 192 × 192 mm^2^, voxel size: 3 × 3 × 3 mm^3^, slice acquisition: interleaved, total 330 measurements). Additionally, T1-weighted anatomical images were obtained using the Magnetisation Prepared Rapid Gradient Echo (MPRAGE) protocol (160 slices, slice thickness: 1 mm, TR/TE: 2300 ms/2.98 ms, flip angle: 9°, FOV: 240 × 256 mm^2^, voxel size: 1 × 1 × 1 mm^3^).

### Neuroimaging data pre-processing

Neuroimaging pre-processing and functional connectivity analysis were performed using the CONN software (https://www.nitrc.org/projects/conn, RRID:SCR_009550) and in-house MatLab scripts (MathWorks, Natrick, MA). The pre-processing sequence in the CONN included motion estimation and correction, correction for inter-slice differences in acquisition time, outlier detection, and segmentation. The anatomical and functional images were coregistered and normalised to a standard brain space (MNI152) and smoothed (8 mm FWHM). Denoising of functional data consisted of white matter and cerebrospinal fluid regression, followed by despiking and band-pass filtering (0.008–0.9 Hz).

Functional images were analysed to identify spatially independent and temporally coherent networks using the Independent Component Analysis (ICA) as described by Calhoun and colleagues [18]. First, a spatial–temporal Principal Component Analysis was performed to reduce fMRI data to a lower dimensionality, which allowed a reduced complexity while persevering most of the information content. A FastICA algorithm [19] with Tahn distribution was used for the estimation of independent spatial components and a GICA1 backprojection [18] was used for individual subject-level spatial map estimation. The number of independent components was set to 17 based on the MDL (i.i.d. sampling) criteria. The optimal number of components was estimated using the Infomax algorithm [20] implemented in the group ICA for fMRI toolbox (GIFT software v4.0b, http://icatb.sourceforge.net). Infomax uses a fixed non-linearity for a super-Gaussian distribution by maximising information transfer between an input and an output of networks. Each component was represented by a spatial map and temporal profile. The resulting maps were used to compute the individual subject components (i.e., back reconstruction of components).

**Fig. 1:**
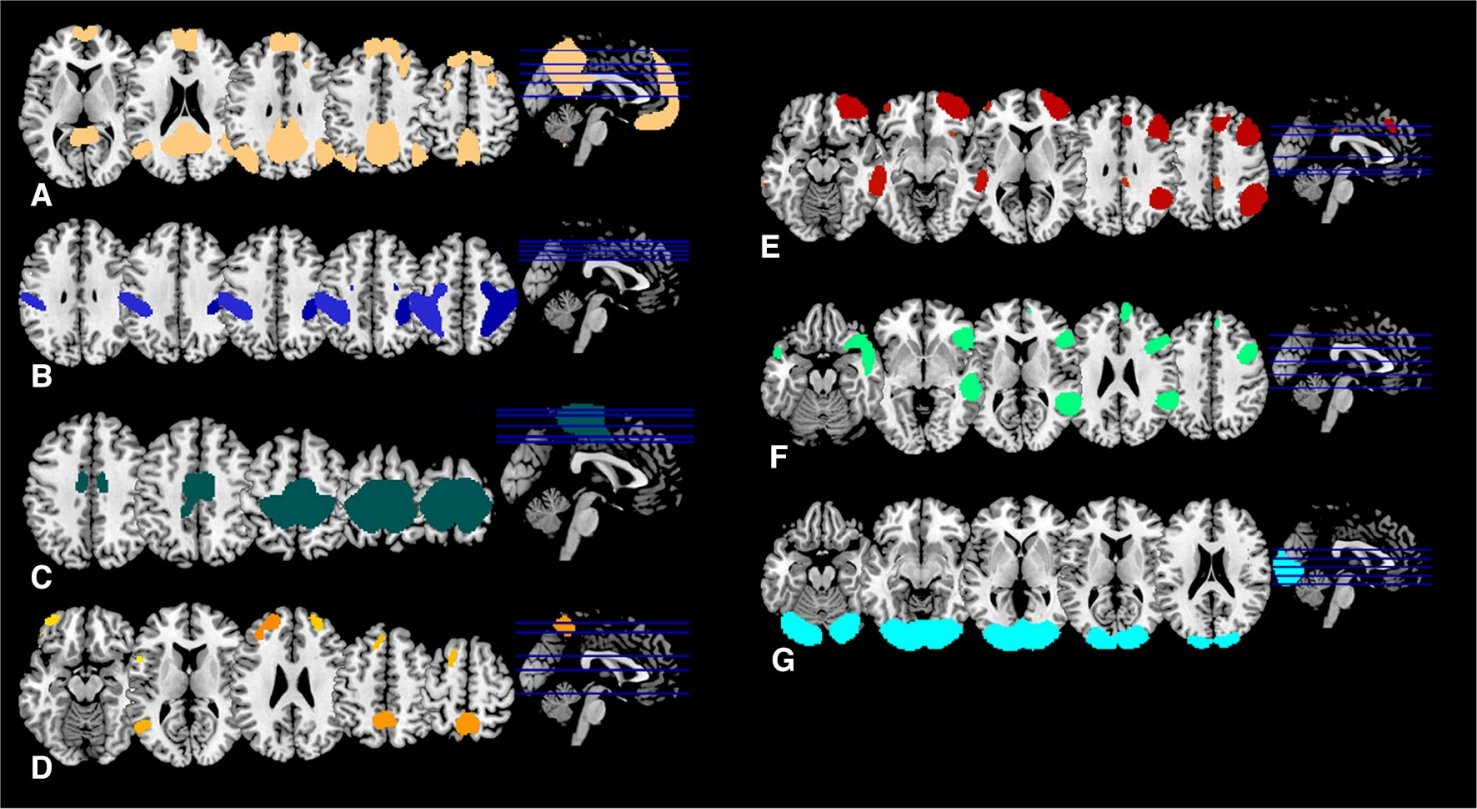
Identified resting-state networks. **A** DMN. **B** Dorsoattentional network. **C** Sensorimotor network. **D** Salience network. **E** Frontoparietal network. **F** Language network. **G** Visual network

The resting-state networks were identified through a spatial correlation across voxels with the predefined connectivity network templates in the CONN, while the components with the highest spatial correlations were selected. This resulted in 7 resting-state networks: the DMN, sensorimotor, visual, salience, dorsoattentional, frontoparietal (i.e., executive network), and language networks (Fig. 1). The visualisation of the spatial correlations with the templates is provided in the Supplementary Material. Notably, both the salience network and the frontoparietal network showed a lower correlation to the network templates than the rest of the networks.

### Between-group analyses

The selected networks were analysed in second-level within-network analyses with groups as factors (*APOEe4* carriers vs non-carriers, *MAPTA* carriers vs non-carriers, and *MAPTG* carriers vs non-carriers) and age, gender, years of education and reported family history of dementia as covariates. Family history of dementia was determined by a self-reported diagnosis of dementia for the participants’ grandparents. We compared resting-state connectivity in all identified resting-state networks between groups by using parametric Gaussian Random Field Theory [21] with cluster FDR-corrected threshold < 0.05 implemented in CONN.

In the next step, seed-based analyses were conducted using the clusters that showed significant between-group differences as seeds. Connectivity values (i.e., calculated as the mean values of time-series) from respective significant clusters were compared with the connectivity values of the rest of the brain, quantifying inter-regional connectivity strength.

To explore the effect of connectivity and genotype on the Stroop task performance, the Stroop performance was calculated as both the accuracy in the colour-incongruent condition, and as the difference between reaction times in colour-incongruent and colour-congruent conditions. Behaviourally, comparisons of Stroop performance between groups were analysed using students’ t-tests. To explore whether Stroop performance varied among individuals as a function of *APOE/MAPT* status interacting with connectivity in each cluster, several linear models were built. In one set of models, Stroop performance was either explained by categorical group status, functional connectivity values in respective clusters, or their interactions. In another set of models, age, gender, education, and family history were additionally used to explain Stroop performance.

## Results

### Within-network and seed-based functional connectivity

**Fig. 2:**
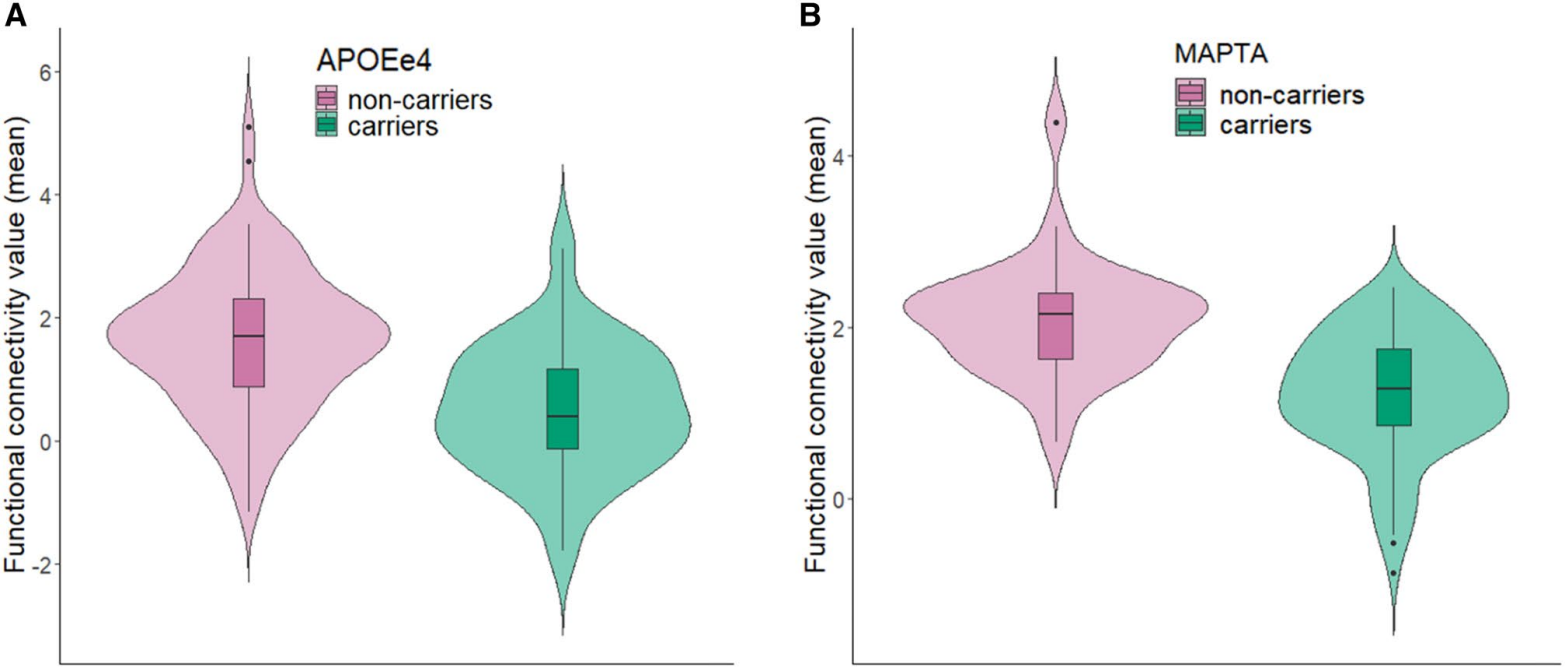
Violin plots of the functional connectivity differences in the DMN calculated based on the peak connectivity values in each cluster. **A** *APOEe4* carriers vs non-carriers in Cluster 1. **B** *MAPTA* carriers vs non-carriers in Cluster 2

**Fig. 3:**
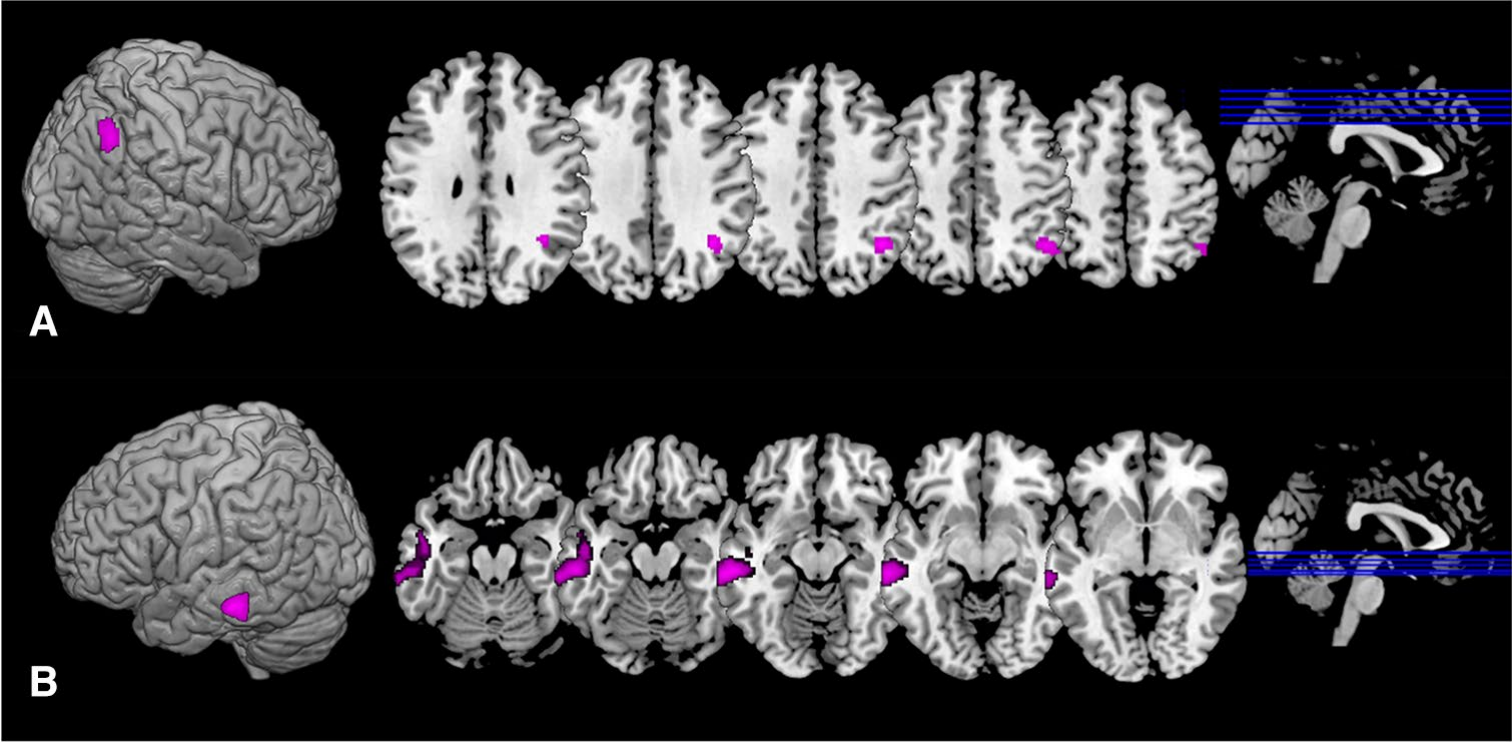
The spatial maps of clusters that exhibited significant differences in connectivity between carriers and non-carriers. **A** Cluster 1 comprising of the right angular gyrus and superior occipital cortex that showed significant differences between *APOEe4* carriers and non-carriers. **B** Cluster 2 comprising of the left middle temporal gyrus that showed significant differences between *MAPTA* carriers and non-carriers

The DMN was the only network that showed significant differences between groups. There was a decreased connectivity in *APOEe4* carriers compared with non-carriers in a cluster that comprised of the right angular gyrus and right superior occipital cortex (Cluster 1; size = 246 voxels, p-FWE = 0.0081, p-FDR = 0.0079; Fig. 2A). Similarly, *MAPTA* carriers showed decreased connectivity in the DMN in comparison with non-carriers in the left middle temporal gyrus cluster (Cluster 2; size = 546 voxels, p-FWE < 0.0001, p-FDR = 0.0001; Fig. 2B). The visual rendering of these clusters is presented in Fig. 3. No statistically significant between-group differences were observed between *MAPTG* -carriers. We additionally controlled for the effect of *APOE* in the *MAPT* analysis and vice versa. The resulting group differences were not changed.

**Fig. 4:**
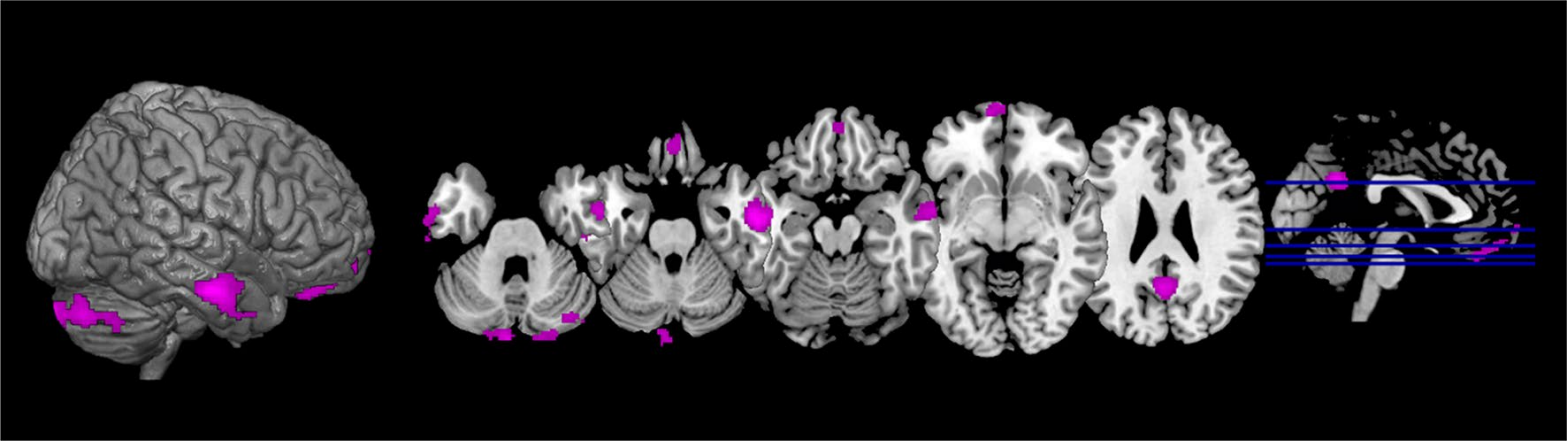
The spatial map of clusters that exhibited an additional reduction of functional connectivity from Cluster 2

The clusters that exhibited significant between-group differences were selected for further seed-based analyses to explore the global connectivity of the rest of the brain with these DMN regions. After assessing for between-group differences, no significant differences in connectivity of Cluster 1 to other brain areas were found between *APOEe4* carriers and non-carriers. However, Cluster 2 exhibited an additional decrease in functional connectivity into multiple areas across the brain in *MAPTA* carriers. Namely, additionally decreased connectivity was observed in the right posterior and anterior middle temporal gyrus (size = 502 voxels, p-FWE = 0.0001, p-FDR = 0.0003), left posterior and anterior middle temporal gyrus (size = 314 voxels, p-FWE = 0.0029, p-FDR = 0.0019), right cerebellum (size = 457 voxels, p-FWE = 0.0002, p-FDR = 0.0003), left cerebellum (size = 222 voxels, p-FWE = 0.0169, p-FDR = 0.0077), precuneus and posterior cingulate cortex (size = 251 voxels, p-FWE = 0.0095, p-FDR = 0.0052), and in left frontal pole and frontal medial cortex (size = 393 voxels, p-FWE = 0.0007, p-FDR = 0.0006). The results are summarised in Table 2, and the visual rendering of all regions that exhibited significant between-group differences in global connectivity with the DMN is shown in Fig. 4.

**Table 2:** Summary of functional connectivity findings in carriers relative to non-carriers. The cluster that showed a significant between-group difference in the DMN functional connectivity (FC) analysis was used as a seed for additional seed-based whole-brain FC analysis

Group	DMN FC	Cluster size (voxels)	Seed-based whole-brain FC	Cluster size (voxels)
*APOEe4*	↓ the angular gyrus and right superior occipital cortex	246	p > 0.05	N/A
*MAPTA*	↓ left posterior middle temporal gyrus	540	↓ right anterior and posterior middle temporal gyrus	502
			↓ right cerebellum	457
			↓ left frontal pole and frontal medial cortex	393
			↓ left anterior and posterior middle temporal gyrus	314
			↓ precuneus and posterior cingulate cortex	251
			↓ left cerebellum	222
*MAPTG*	p > 0.05	N/A	N/A	N/A

### Stroop performance affected by functional connectivity and genotypes

Eleven participants were excluded from the analysis due to not completing the cognitive tasks. Two of those participants were *APOEe4* carriers and eight were *MAPTA* carriers.

The between-group analysis of the performance on the Stroop task did not reveal any significant differences in accuracy in the colour-incongruent condition between the *APOEe4* carriers and non-carriers (t(116) = 0.44, p > 0.05), *MAPTA* carriers and non-carriers (t(116) = − 0.03, p > 0.05), or *MAPTG* carriers and non-carriers (t(116) =  − 0.01, p > 0.05). Similarly, the analysis did not reveal any significant differences when Stroop performance was calculated as the difference between reaction times in colour-incongruent and colour-congruent conditions (*APOEe4* carriers and non-carriers (t(116) =  − 0.62, p > 0.05), *MAPTA* carriers and non-carriers (t(116) = 0.56, p > 0.05), and *MAPTG* carriers and non-carriers (t(116) =  − 0.16, p > 0.05)).

We were further interested in exploring whether Stroop performance (i.e., accuracy in the colour-incongruent condition) varied among individuals as a function of *APOE/MAPT* status interacting with connectivity in each cluster. Several linear models were built in which Stroop performance was either explained by categorical group status, functional connectivity values in respective clusters, or their interactions.

After fitting the models for *APOE*, we observed that the model that contained the interaction between *APOE* status and connectivity in Cluster 1 explained the Stroop task performance significantly better than the model without the interaction (RSS = 0.221, sum of sq = 0.011, F = 5.787, p = 0.018). Specifically, we observed significant effects of *APOE* status (t = − 2.181, p = 0.031), connectivity in Cluster 1 (t = − 2.232, p = 0.028), and their interaction on Stroop performance (t = 2.417, p = 0.017). Neither age, gender, education, nor family history improved the performance of the model (p > 0.05). The interaction suggested that with any additional decrease in connectivity, there is a decrease in Stroop performance for *APOEe4* carriers (coefficient estimate for the interaction = 0.024, p = 0.015).

**Fig. 5:**
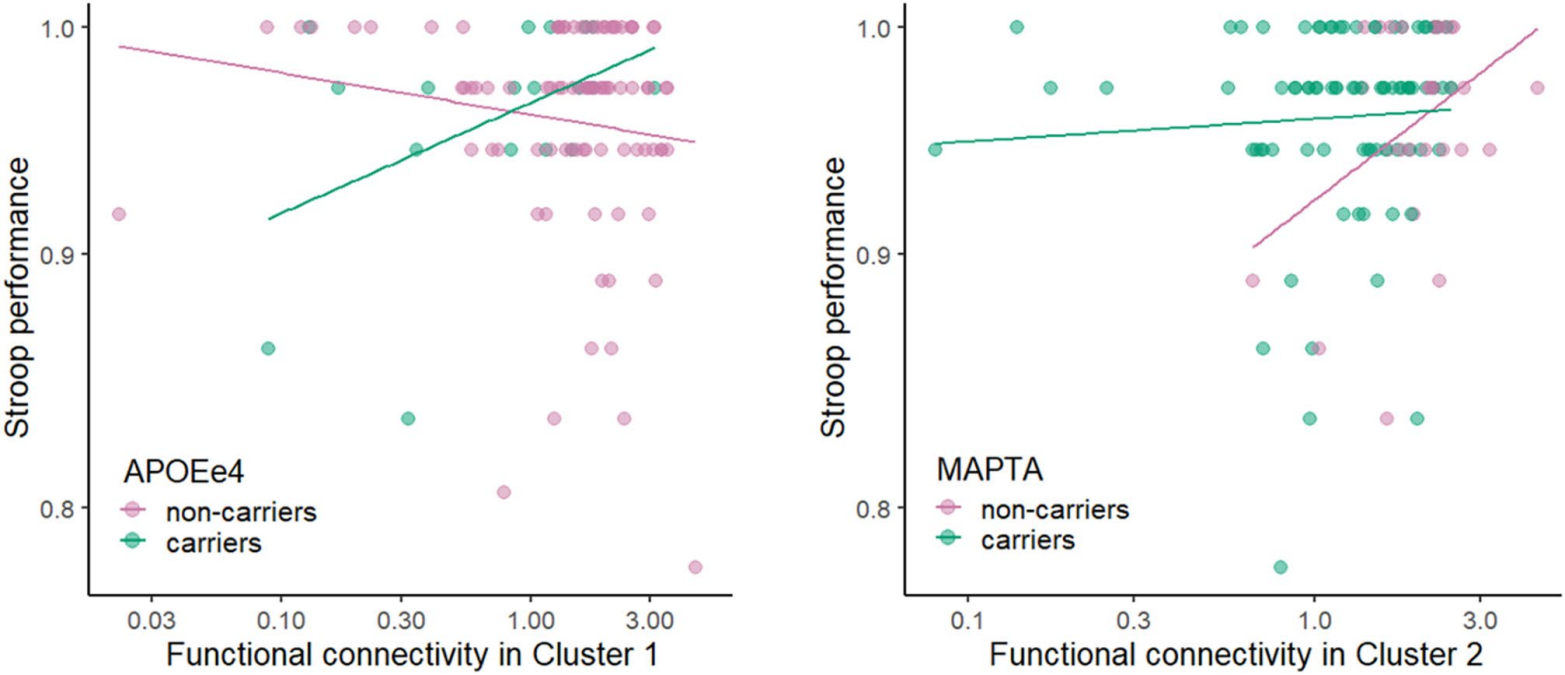
Spearman’s correlations between functional connectivity in respective clusters and Stroop performance (i.e., the accuracy in the colour-incongruent condition) between groups with fitted linear regression lines. *APOEe4* carriers showed a positive relationship between connectivity in Cluster 1 and Stroop performance (R = 0.42, p = 0.037), while no other relationship was observed in *APOEe4* non-carriers (R = − 0.16, p > 0.05), *MAPTA* carriers (R = 0.08, p > 0.05), and *MAPTA* non-carriers (R = 0.2, p > 0.05)

After fitting *MAPT* models, the performance of the models did not differ significantly (p > 0.5). Adding age, gender, education, or family history did not improve the performance of the model (p > 0.05). These results were in line with the results observed by using Spearman’s correlation of Stroop performance and DMN connectivity (Fig. 5).

We further repeated the analyses by calculating Stroop performance as the difference in reaction times between the conditions (i.e., colour-incongruent and colour-congruent). The Stroop performance did not vary as a function of the *APOE* or *MAPT*, connectivity, or their interaction (p > 0.05). Neither age, gender, education, nor family history improved the performance of the model (p > 0.05).

## Discussion

The present study used ICA and Gaussian Random Theory to study functional connectivity differences in carriers of genetic risk factors for AD. Our results indicated decreased functional connectivity in the DMN in the angular gyrus and lateral occipital areas in *APOEe4* carriers in comparison with non-carriers. Comparing the inter-regional connectivity strength of this area with that of the rest of the brain did not reveal any additional disruptions of global brain connectivity. Compared to *MAPTA* non-carriers, *MAPTA* carriers displayed decreased functional connectivity in the DMN in the left middle temporal gyrus with additional disruptions in global brain connectivity in the areas that are often linked with AD-related pathology (e.g., precuneus, posterior cingulate cortex, and right middle temporal gyrus). Moreover, we observed a relationship between connectivity and cognition in *APOEe4* carriers which was not observed in *MAPTA* carriers. This could suggest different underlying mechanisms of the genetics-related vulnerability of the brain.

In previous research, young *APOEe4* carriers showed consistent functional connectivity disturbances in the DMN; yet, the directionality of the findings is inconclusive in the literature. For instance, studies with a methodology similar to that of the current study showed contradicting increased connectivity in young *APOEe4* carriers in multiple DMN areas [22–24]. One of the factors that could add to the observed discrepancy between our findings and available literature could be the younger age of our participants (mean age: 19.6 years) than that in studies with similar methodology (21.4 years, 28.6 years, 23.8 years, respectively). Although not notably large, this difference might be crucial due to the evidence of large-scale network stabilisation that continues until early adulthood [25] and may differ between sexes [26]. In our study, we did not run additional analyses based on gender as this would result in insufficient power in some groups, especially in *APOEe4* carriers. However, when using gender as one of the covariates in the models, the performance did not change significantly.

A systematic review of MRI-related brain changes also revealed that there is generally only limited evidence covering the age range between 18 and 21 years [27], which makes comparisons with other functional neuroimaging changes in the DMN regions additionally challenging. For instance, there are only two recent functional neuroimaging studies with a mean age of *APOEe4* adult carriers below 20 years. They reported increased functional connectivity in the posterior regions of the DMN [28] and in the medial temporal regions [29] while using the same sample and a discrimination task.

There are two notable differences between our sample and those of the aforementioned studies. First, the total sample size of our study (n = 129) was larger than that of other similar studies. To our knowledge, there are only two resting-state studies investigating the effect of genetic risk factors on functional connectivity with a similar [30] or a larger total sample size [31]. The mean ages of *APOEe4* carriers in these studies are 28.8 and 22.8 years respectively. Using different methodologies, both studies yielded mixed results. While there was no difference in the functional connectivity between young *APOEe4* carriers and non-carriers, [30], there was both a decrease and increase in connectivity between various brain areas in *APOEe4*-*KIBRA* carriers [31].

Another difference is linked to the ethnicity of the participants. Previous research suggests that *APOEe4* may modulate DMN connectivity differently in different ethnicities [32]. While the ethnic background of the participants in studies with contradictory findings is unclear, the majority recruited their participants in the UK [22, 28, 29]. It can be assumed that their ethnicity differed from that of the Chinese Han. Nevertheless, there were only two other resting-state functional connectivity studies with ethnic Chinese Han samples [30, 31]. The findings of the two studies are discussed above.

Interestingly, no previous studies in at-risk young populations indicated considerable connectivity differences in the angular gyri. However, decreased functional connectivity in these areas was observed in converters with Mild Cognitive Impairment [33]. The structures of the angular gyri have been also previously linked with energy metabolism changes that could predict a decline in global cognitive function [34]. We also observed a moderate relationship between the performance on the Stroop task and a decline in functional connectivity in the right angular gyrus cluster when calculating the Stroop performance as the accuracy in the colour-incongruent condition. This relationship indicated that with decreasing functional connectivity, the accuracy in the colour-incongruent condition in the Stroop task additionally worsened. One potential explanation for this relationship is the binding role of the angular gyrus between different domains and processing streams (e.g., visuospatial, somatosensory, auditory) that are involved in multiple cognitive functions [35]. Reduced connectivity in these areas may compromise this binding function, resulting in reduced accuracy in the Stroop task.

Notably, when calculating Stroop performance as the reaction time difference between the conditions, we did not observe this relationship. Such discrepancy has been observed in multiple different paradigms such as tactile decision-making [36, 37] and could be explained by the employment of different cognitive and neural mechanisms.

Reduced functional connectivity in individuals with *MAPT* genetic risk factors in the medial temporal areas is mostly observed in studies targeting behavioural variants of frontotemporal dementia [38]. Little is known about the relationship between functional connectivity and the *MAPT* gene in patients with AD. Some evidence from animal models of early-stage AD suggests functional connectivity disturbances in the medial temporal regions [39]. *AppNL-F/MAPT* double knock-in mice displayed disturbances in brain network organisation within the medial temporal regions and an additional global reduction of connectivity to other brain regions, which is in line with our results. The limited empirical evidence of the relationship between *MAPT*, AD, and connectivity disturbances should be taken into consideration when interpreting the present results. This is an important issue for future research that should consider a wider range of genetic and environmental risk factors of AD beyond the *APOEe4* allele to answer further questions about the underlying mechanisms of related connectivity changes. GWAS and polygenic risk score studies could also be important additions to more efforts in personalised medicine.

Intriguingly, there was no significant difference in functional connectivity between *MAPTG* carriers and non-carriers. This suggests that grouping the AG allele with other genotypes has a significant impact on the results. We recommend considering this in future studies, similar to accounting for the impact of ethnicity on genetic risk.

The limitations of the present study relate to technical aspects of the analytical toolbox of choice (CONN) which does not provide the network stability assessment (e.g., by using the ICASSO algorithm). Additionally, it does not provide the correlation values of the spatial correlation of independent components with the network templates beyond the visualisation. This makes the stability and reliability assessment of the identified networks difficult and should be taken into account. For instance, the salience network and the frontoparietal network show lower correlations with the templates in comparison to other networks in the study which should be carefully considered when interpreting the results.

## Conclusion

Our findings offer an important insight into the functional connectivity patterns across the lifespan, specifically in young adulthood, while adding to the limited evidence of the role of genetic risk factors for AD in functional brain changes. Whilst *MAPTA* carriers demonstrated more global disturbances in functional connectivity, *APOEe4* carriers showed a link between functional connectivity and cognition. Hence, our results suggest that while both *APOEe4* and *MAPTA* alleles modulate brain functional connectivity in young adults, they may do so via different underlying mechanisms.

### Supplementary Information

Below is the link to the electronic supplementary material.

Supplementary file1 (DOCX 357 KB)
