# Supplementary material for: Genetic risk factors of Alzheimer’s Disease disrupt resting-state functional connectivity in cognitively intact young individuals
Source: J Neurol. 2023 Jun 26;270(10):4949–58. doi: 10.1007/s00415-023-11809-9 (PMC10511575; doi:10.1007/s00415-023-11809-9)

Spatial maps of identified independent components (17) and the visualisation of the correlations with the network templates as provided by CONN toolbox:


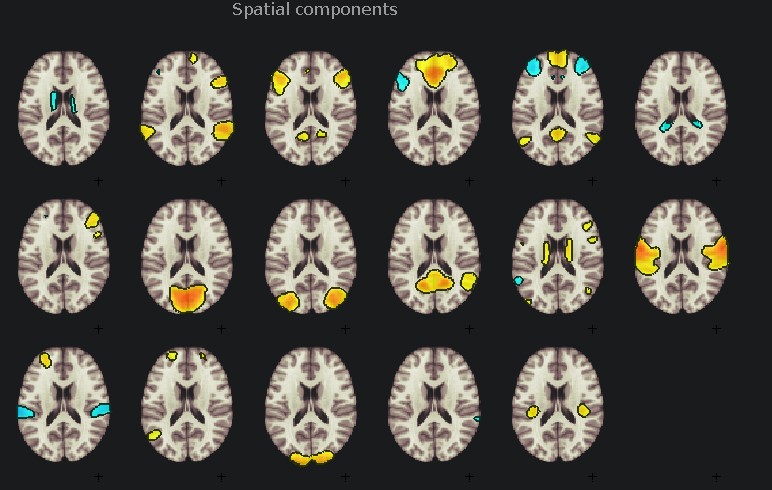


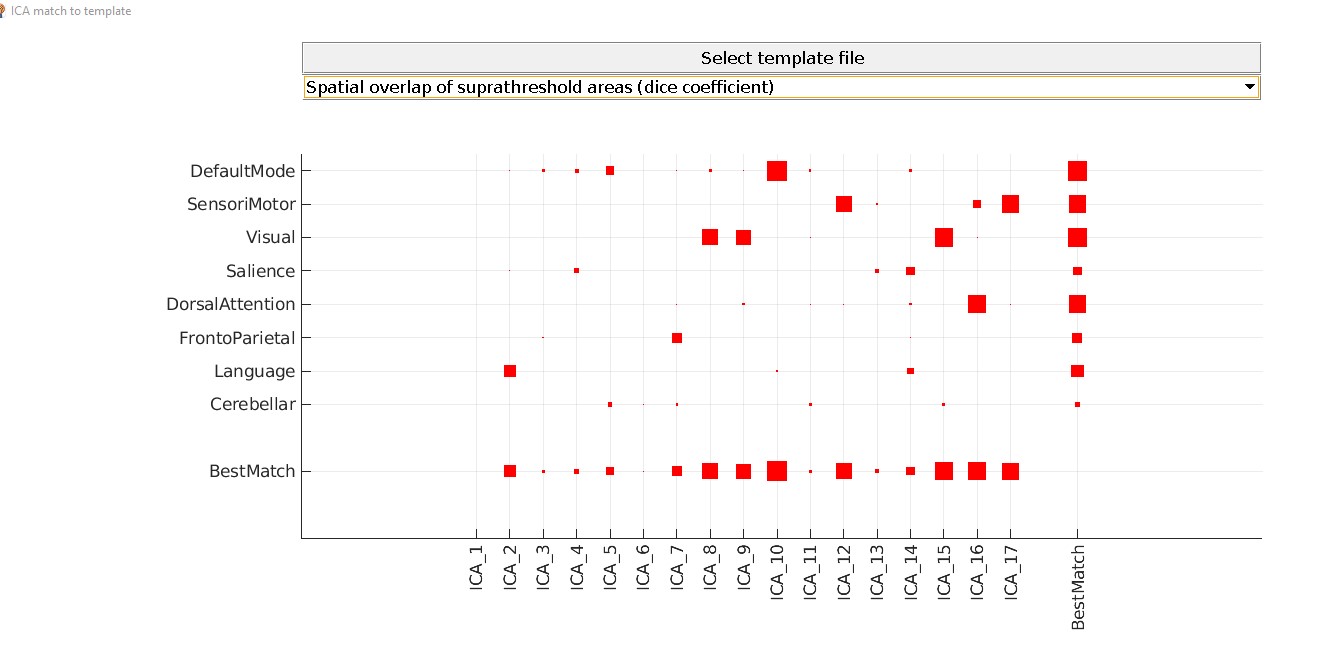

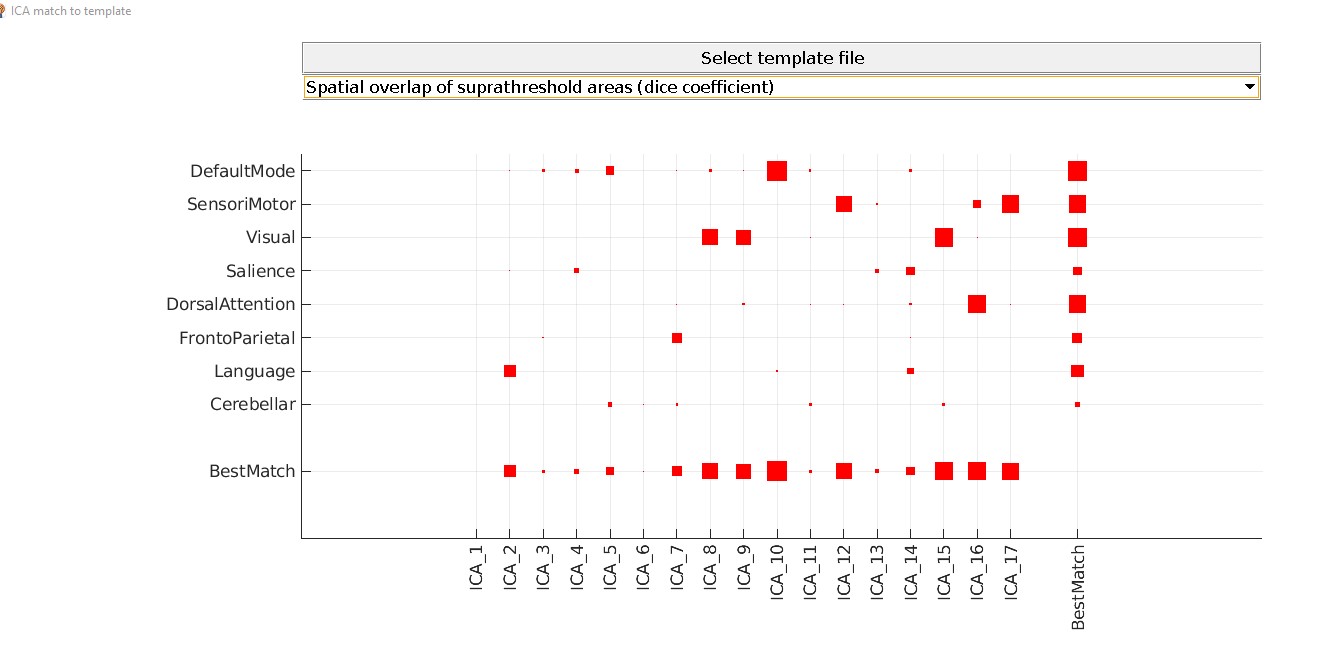

Supplement: Supplementary file 1 — Supplementary file1 (DOCX 357 KB) [file 415_2023_11809_MOESM1_ESM.docx]
